# Mechanical stimulation promotes fibrochondrocyte proliferation by activating the TRPV4 signaling pathway during tendon–bone insertion healing: CCN2 plays an important regulatory role

**DOI:** 10.1093/burnst/tkae028

**Published:** 2024-10-19

**Authors:** Xuting Bian, Xiao Liu, Mei Zhou, Hong Tang, Rui Wang, Lin Ma, Gang He, Shibo Xu, Yunjiao Wang, Jindong Tan, Kanglai Tang, Lin Guo

**Affiliations:** State Key Laboratory of Trauma, Burn and Combined Injury, Department of Orthopedics/Sports Medicine Center, First Affiliated Hospital of Army Medical University, No. 30 Gaotanyan Street, Shapingba District, Chongqing, 400038, China; State Key Laboratory of Trauma, Burn and Combined Injury, Department of Orthopedics/Sports Medicine Center, First Affiliated Hospital of Army Medical University, No. 30 Gaotanyan Street, Shapingba District, Chongqing, 400038, China; State Key Laboratory of Trauma, Burn and Combined Injury, Department of Orthopedics/Sports Medicine Center, First Affiliated Hospital of Army Medical University, No. 30 Gaotanyan Street, Shapingba District, Chongqing, 400038, China; State Key Laboratory of Trauma, Burn and Combined Injury, Department of Orthopedics/Sports Medicine Center, First Affiliated Hospital of Army Medical University, No. 30 Gaotanyan Street, Shapingba District, Chongqing, 400038, China; Chongqing Institute of Bio-Intelligent Manufacturing, No. 60, Xingguang Avenue, Yubei District, Chongqing, 400000, China; State Key Laboratory of Trauma, Burn and Combined Injury, Department of Orthopedics/Sports Medicine Center, First Affiliated Hospital of Army Medical University, No. 30 Gaotanyan Street, Shapingba District, Chongqing, 400038, China; State Key Laboratory of Trauma, Burn and Combined Injury, Department of Orthopedics/Sports Medicine Center, First Affiliated Hospital of Army Medical University, No. 30 Gaotanyan Street, Shapingba District, Chongqing, 400038, China; State Key Laboratory of Trauma, Burn and Combined Injury, Department of Orthopedics/Sports Medicine Center, First Affiliated Hospital of Army Medical University, No. 30 Gaotanyan Street, Shapingba District, Chongqing, 400038, China; State Key Laboratory of Trauma, Burn and Combined Injury, Department of Orthopedics/Sports Medicine Center, First Affiliated Hospital of Army Medical University, No. 30 Gaotanyan Street, Shapingba District, Chongqing, 400038, China; State Key Laboratory of Trauma, Burn and Combined Injury, Department of Orthopedics/Sports Medicine Center, First Affiliated Hospital of Army Medical University, No. 30 Gaotanyan Street, Shapingba District, Chongqing, 400038, China; State Key Laboratory of Trauma, Burn and Combined Injury, Department of Orthopedics/Sports Medicine Center, First Affiliated Hospital of Army Medical University, No. 30 Gaotanyan Street, Shapingba District, Chongqing, 400038, China; State Key Laboratory of Trauma, Burn and Combined Injury, Department of Orthopedics/Sports Medicine Center, First Affiliated Hospital of Army Medical University, No. 30 Gaotanyan Street, Shapingba District, Chongqing, 400038, China

**Keywords:** Mechanical stimulation, Fibrochondrocyte, Proliferation, Tendon–bone insertion healing, TRPV4, CCN2, Cartilage repair, PI3K/AKT signaling pathway

## Abstract

**Background:**

We previously confirmed that mechanical stimulation is an important factor in the repair of tendon–bone insertion (TBI) injuries and that mechanoreceptors such as transient receptor potential ion-channel subfamily V member 4 (TRPV4; also known as transient receptor potential vanilloid 4) are key to transforming mechanical stimulation into intracellular biochemical signals. This study aims to elucidate the mechanism of mechanical stimulation regulating TRPV4.

**Methods:**

Immunohistochemical staining and western blotting were used to evaluate cartilage repair at the TBI after injury. The RNA expression and protein expression of mechanoreceptors and key pathway molecules regulating cartilage proliferation were analyzed. TBI samples were collected for transcriptome sequencing to detect gene expression. Calcium-ion imaging and flow cytometry were used to evaluate the function of TPRV4 and cellular communication network factor 2 (CCN2) after the administration of siRNA, recombinant adenovirus and agonists.

**Results:**

We found that treadmill training improved the quality of TBI healing and enhanced fibrochondrocyte proliferation. The transcriptome sequencing results suggested that the elevated expression of the mechanistically stimulated regulator CCN2 and the exogenous administration of recombinant human CCN2 significantly promoted TRPV4 protein expression and fibrochondrocyte proliferation. *In vitro*, under mechanical stimulation conditions, small interfering RNA (siRNA)-CCN2 not only inhibited the proliferation of primary fibrochondrocytes but also suppressed TRPV4 protein expression and activity. Subsequently, primary fibrochondrocytes were treated with the TRPV4 agonist GSK1016790A and the recombinant adenovirus TRPV4 (Ad-TRPV4), and GSK1016790A partially reversed the inhibitory effect of siRNA-CCN2. The phosphoinositide 3-kinase/ protein kinase B (PI3K/AKT) signaling pathway participated in the above process.

**Conclusions:**

Mechanical stimulation promoted fibrochondrocyte proliferation and TBI healing by activating TRPV4 channels and the PI3K/AKT signaling pathway, and CCN2 may be a key regulatory protein in maintaining TRPV4 activation.

## Background

Tendon–bone insertion (TBI) healing is a difficult problem in the field of bone and joints. Due to its limited blood supply and unique tissue structure, the tissue composition in the narrow region changes from tendon to fibrocartilage and calcified fibrocartilage and eventually transits to bone tissue, with adaptive changes occurring in cells, the extracellular matrix and gene expression throughout this location, resulting in a limited ability to repair itself [1–3]. Clinical studies have indicated that the mechanical environment originally maintained by the mineral gradient of the fibrocartilage complex is completely destroyed after TBI injury, and despite significant improvements in surgical fixation, the mechanical microenvironment cannot be fully restored after surgical repair due to the complexity of the mechanical and tissue levels and the large amount of scar tissue filling the injury site, which ultimately leads to the failure of structural reconstruction of the new fibrocartilage complex [4, 5]. Many studies have confirmed that mechanical stimulation is essential for TBI healing and that moderate mechanical stimulation can change the local physical microenvironment to promote healing [6, 7]. Sports rehabilitation training is also necessary to promote the postoperative rehabilitation of patients with TBI injury [8], but the molecular basis of mechanical stimulation for TBI healing is still not fully understood.

Chondrocytes are the main effector cells that sense mechanical stimulation during TBI healing. Mechanical stimulation is transmitted to chondrocyte membrane mechanoreceptors through extracellular matrix-mediated transmission, which eventually converts mechanical stimulation into intracellular biochemical signals and regulates target gene expression and cellular behavior [9]. Common mechanoreceptors include Ca^2+^ channels [10, 11], integrin signals [12, 13] and primary cilia [14]. Transient receptor potential vanilloid 4 (TRPV4; also known as transient receptor potential ion-channel subfamily V member 4) is a nonselective cation channel permeable to Ca^2+^ that is highly expressed in chondrocytes, primarily senses chondrocyte mechanical signals and is an important molecule in the regulation of chondrogenesis and homeostasis [15, 16], and its loss of function is closely associated with a variety of cartilage diseases [17, 18]. Du G *et al*. found that TRPV4 was primarily responsible for sensing mechanical stimulation at the physiological level [19]. Willard *et al*. recently showed that TRPV4 was specifically expressed in the chondrogenic cell population of the collagen type II alpha 1 (Col2a1)-green fluorescent protein (GFP) reporter gene, while dynamic TRPV4 activation promoted cartilage extracellular matrix synthesis and chondrogenic gene expression, suggesting that TRPV4 could act both as a marker and a regulator of chondrocyte formation [16]. The extracellular matrix can act as a nonlinear mechanical adapter to provide chondrocytes with a reduced mechanical stress gradient [20]. In addition, the interaction of the extracellular matrix with mechanoreceptors is also an important mechanism by which chondrocytes perceive mechanical stimulation [21]; however, it is unclear whether TRPV4 exerts its function while being regulated by extracellular matrix proteins.

Cellular communication network factor 2 (CCN2) is an important component of the extracellular matrix and a regulator of mechanical signaling in chondrocytes, and mechanical signaling is also an important factor in elevated CCN2 expression. Hara *et al*. found that CCN2 could act synergistically with other cytokines to promote damaged HCS-2/8 cartilage regeneration and extracellular matrix synthesis, suggesting that CCN2 could act as a mesenchymal tissue regenerating agent [22]. A recent study by Nishida and Kubota suggested that CCN2 could act as a regulator of mechanical signaling in chondrocytes, with elevated expression under appropriate mechanical stress, and could be involved in regulating biological behaviors such as chondrocyte proliferation and chondrogenesis [23]. Experimental studies have shown that CCN2 interacting with integrin α5 promoted chondrocyte proliferation by activating the extracellular regulated protein kinases (ERK) pathway [24]. Basic studies have shown that TRPV4 was specifically highly expressed in chondrocytes during TBI healing and acted as a major mechanoreceptor for sensing mechanical stimulation [25]; however, whether CCN2 can influence chondrocyte biological behavior through TRPV4 modulation to promote TBI healing is unclear.

Elucidating the mechanism by which mechanical stimulation promotes TBI healing is essential for the development of new physical or pharmacological treatments. We hypothesize that CCN2 may facilitate the conversion of mechanical into biochemical signaling processes in fibrochondrocytes by affecting TRPV4 activity under mechanical stimulation, thereby enhancing fibrochondrocyte proliferation and the quality of TBI healing.

## Methods

### Animal model and treadmill training

Eight-week-old male C57BL/6 mice (Byrness Weil Biotech Ltd, Chongqing, China) were used in this study. The animal experimental protocols were reviewed and approved by the Animal Ethics Committee of the Army Medical University. We established a mouse model of Achilles TBI injury. Briefly, anesthesia was induced by intraperitoneal injection of 0.3% sodium pentobarbital (0.1 ml/10 g) into the right lower abdomen, and the Achilles TBI was severed with a scalpel. The surface cartilage layer was removed and a bone tunnel was made through the bone ~2 mm below the surface of the Achilles bone with a bone marrow tract needle to facilitate the passage of sutures to reattach the Achilles tendon to the surface of the heel bone [26]. All the mice were allowed to move freely in the cage after surgery. Mice in the treadmill training group were started on postoperative day 7after surgery with treadmill parameters of 10 m/min, 30 min/d, 5 d/week, and a 0° inclination angle [27]. Mice in the recombinant human CCN2 (rhCCN2) group were treated with a local injection of 0.5 μg of rhCCN2 (MedChemExpress, USA) at the TBI once a week [28–30]. All the mice were sacrificed by neck amputation at 3 months after surgery, and specimens were subsequently collected.

### Fibrochondrocyte isolation and culture

Primary fibrochondrocytes were extracted from the Achilles TBI of mice. In brief, the cartilage tissue was cut into 2-mm blocks, added to serum-free medium containing 0.5% trypsinase, and subjected to continued enzymatic digestion for 1 h. Following this, the medium was substituted with serum-free medium, containing 0.1% hyaluronidase and 0.5% type II collagenase, and incubated at 37°C for 2–3 h with agitation. The resulting solution was then passed through a 40 μm filter to remove debris, followed by centrifugation at 500 g for 5 min to isolate the primary fibrochondrocytes. These primary fibrochondrocytes were seeded into a 75 cm^2^ cell culture flask containing Dulbecco’s modified Eagle’s medium supplemented with 10.0% fetal bovine serum and 1% penicillin–streptomycin and maintained in a 37°C incubator with 5.0% CO_2_. The culture medium was refreshed every 1–2 days and fibrochondrocytes of the P2 generation were utilized for subsequent experiments.

### Mechanical stimulation

Fibrochondrocytes were seeded into a stretch chamber (NEPA GENE, Japan) coated with fibronectin at a density of 5 × 10^4^ cells per well within a Forma Series II Water Jacketed CO_2_ Incubator (Thermo Fisher Scientific, USA) maintained at 37°C under a 5.0% CO_2_ atmosphere. Upon reaching a cell density of 70–80%, uniaxial cyclic stretch experiments were conducted using a uniaxial cyclic stretch system (8.0% elongation in length, 1 Hz) for 8 h. The control group underwent identical conditions, excluding the uniaxial cyclic stretching component.

### Fibrochondrocyte siRNA treatment

CCN2 siRNA and negative control (NC) siRNA were designed and synthesized by Shanghai GenePharma Co., Ltd (Shanghai, China). The designed siRNA-CCN2 sequences were 5′-GCACCAGUGUGAAGACAUATT-3′ (sense) and 5′-UAUGUCUUCACACUGGUGCTT-3′ (antisense). Fibrochondrocytes were spread in a stretch chamber and cultured with 1.6 ml of serum-free medium. Then, 8 μl of siRNAs and 4 μl of GP-transfect-Mate (Zeta Life, USA) were mixed with 200 μl of serum-free medium and left for 5 min. Both were mixed and left for 15–20 min, followed by the addition of 400 μl of transfection mixture to the stretch chamber for a final volume of 2 ml, which was later replaced with complete medium after 4–6 h. Uniaxial cyclic stretching experiments were performed after 48 h of incubation.

### TRPV4 overexpression via recombinant adenovirus transfection

To construct TRPV4-overexpressing fibrochondrocytes, recombinant adenovirus carrying TRPV4 (Ad-TRPV4) and a TRPV4 mimic vector (Ad-NC) were purchased from Shanghai Genechem Co., Ltd (Shanghai, China). In brief, fibrochondrocytes were inoculated into 6-well plates at a cell density of 30–50% and infected with Ad-TRPV4 or Ad-NC for 48 h. Fluorescence microscopy and western blot assays were used to observe the transfection efficiency of Ad-TRPV4 (Figure S1a, b, see online supplementary material), in which the molecular weight of TRPV4-GFP is ~100 kDa.

### TRPV4 activation

The complete medium was replaced with Dulbecco’s modified Eagle’s medium + 2 mmol/l glutamine (without serum) overnight, followed by the addition of 10 nmol of the TRPV4 agonist GSK1016790A (Med Chem Express, USA) to the stretch chamber inoculated with fibrochondrocytes, and an equal amount of dimethyl sulfoxide was added as a control.

### Ca^2+^ signaling measurement

A working solution of Fluo-4 AM (Med Chem Express, USA) at a concentration of 1 μmol/l was applied to a glass-bottomed cell culture dish (Biosharp, Beijing, China). The dish was then incubated in a CO_2_ incubator at 37°C with 5.0% CO_2_ for 30 min, after which the Fluo-4 AM working solution was carefully removed. The cells were washed with Hanks' balanced salt solution (HBSS) solution and then incubated with HBSS solution for 30 min, and imaging was performed using a Zeiss LSM 510 laser scanning confocal microscope (Zeiss, Germany). The mean fluorescence intensity was analyzed using ImageJ analysis software.

### Real-time polymerase chain reaction

The real-time polymerase chain reaction (RT-PCR) procedure used was similar to that used in our previous report [31]. In brief, total RNA was extracted from the samples using TRIzol reagent (Invitrogen, USA), reverse transcription was performed with a cDNA synthesis kit containing PrimeScript™ RT Master Mix (Takara, Japan) and RT-PCR was performed with a CFX96 Touch real-time fluorescence quantitative PCR instrument (Bio-Rad, USA) and SYBR Green RT-PCR kit (Takara, Japan). The relative expression of the target gene, glyceraldehyde 3-phosphate dehydrogenase, was calculated using the 2^−ΔΔCt method. The primer sequences are shown in supplementary Table S1 (see online supplementary material).

### Western blot

Tissue and cellular protein extraction was performed with T-PER tissue protein extraction reagent (Thermo Fisher Scientific Inc., USA). The total protein concentration was determined using a bicinchoninic acid (BCA) protein analysis kit (Beyotime, China). Sodium dodecyl sulfate - polyacrylamide gel electrophoresis (SDS-PAGE) protein loading buffer (5X) (Beyotime, China) was added at a volume ratio of 1 : 4 and the samples were then heated at 95°C for 10 min. The sample protein loading volume was adjusted according to the total protein concentration, and the proteins were separated by protein electrophoresis using a 10% SDS-polyacrylamide gel and transferred onto polyvinylidene difluoride membranes (Merck Millipore Ltd, Germany). The membranes were blocked in 5% nonfat milk containing 0.1% tris buffered saline (TBS)-Tween for 1 h at room temperature and incubated with the primary antibody overnight in a 4°C refrigerator. The primary antibodies used were as follows: rabbit anti-CCN2 (1 : 1000, ab6992, Abcam), rabbit anti-TRPV4 (1 : 1000, PA5–77319, Invitrogen), rabbit anti-Col2a1 (1 : 1000, ab34712, Abcam), rabbit anti-SRY-box transcription factor 9 (Sox9) (1 : 1000, ab185966, Abcam), rabbit anti-aggrecan (Acan; 1 : 1000, NB100–74350, Novus), rabbit anti-phosphoinositide 3-kinase (PI3K) (1 : 1000, ab191606, Abcam), rabbit anti-phosphorylated PI3K (p-PI3K) (Y607) (1 : 1000, ab182651, Abcam), rabbit anti-protein kinase B (AKT) (1 : 1000, 10 176–2-AP, Proteintech), rabbit anti-phosphorylated AKT (p-AKT) (Ser473) (1 : 1000, 28 731–1-AP, Proteintech) and rabbit anti-β-actin (1 : 5000, 81 115–1-RR, Proteintech). The next day, the protein bands were incubated with goat anti-rabbit IgG (H&L)-horseradish peroxidase conjugate (1 : 2000, SA00001–2, Proteintech) for 2 h at room temperature, and the protein bands were detected using a Super ECL western blotting detection kit (Advansta, USA). The protein bands were analyzed semiquantitatively using Image-Pro Plus 5.1 analysis software.

### Histological staining and histomorphological scoring

The specimens were fixed in 4.0% paraformaldehyde for 2 d, decalcified in ethylenediaminetetraacetic acid (EDTA) decalcifying solution for 2 d, and ultimately dehydrated in sucrose–paraformaldehyde for 2 d. The specimens were sectioned longitudinally to a thickness of 7 μm. The sections were stained with hematoxylin–eosin (Solarbio, China), and the TBI healing scoring system described by Ide *et al*. was modified for scoring, with a total score of 24 (Table S2, see online supplementary material) [32]. A higher score indicates better TBI healing maturity.

### Immunohistochemical staining

Frozen sections were rewarmed in an incubator for 30 min at 60°C, rehydrated, incubated in an endogenous peroxidase blocker at room temperature for 10 min, washed with PBS three times and incubated with drops of normal goat serum working solution at room temperature for 10 min. Primary antibodies were added and the sections were incubated overnight at 4°C in the refrigerator. The following primary antibodies were used: rabbit anti-CCN2 (1 : 500, ab6992, Abcam), rabbit anti-TRPV4 (1 : 500, PA5–77319, Invitrogen), rabbit anti-Col2a1 (1 : 500, ab34712, Abcam) and rabbit anti-Ki67 (1 : 500, 28 074–1-AP, Proteintech). The sections were washed three times with PBS for 5 min each time, and horseradish peroxidase-conjugated secondary antibody polymer was added and incubated at 37°C for 2 h. Then, chromogenic DAB (3,3’-diaminobenzidine) and hematoxylin were used to stain the nuclei. Finally, the sections were mounted using neutral balsam.

### Bromodeoxyuridine assay and annexin-V/propidium iodide staining

Proliferation assays were performed using a bromodeoxyuridine (BrdU) cell proliferation assay kit (BD Biosciences, USA). In brief, primary fibrochondrocytes were grown in a stretch chamber, and 1 μl of BrdU antibody was added to each stretch chamber 4 h prior to formal cell processing. The cells were fixed, lysed and subsequently incubated in a dark environment for 20 min with added anti-BrdU antibody. The cells were then resuspended in 200 μl of PBS and transferred to 75 mm flow tubes for flow cytometry detection. Apoptosis was detected using an annexin fluorescein 5-isothiocyanate (V-FITC)/propidium iodide double-stained apoptosis detection kit (BestBio, China), and the cells were collected and transferred to 75 mm flow tubes for annexin V-FITC and propidium iodide labeling, followed by flow cytometry to determine the apoptosis rate. The results were analyzed using FlowJo 10.8.1 software.

### Statistical analysis

All quantitative data in our study are presented as the mean ± standard deviation (SD). When the data were normally distributed, one-way analysis of variance (ANOVA), two-way ANOVA and independent t tests were used. *P* < 0.05 indicated a statistically significant difference. The *post hoc* test for ANOVA in this study was least significance difference (LSD). SPSS 25.0 (SPSS, Inc., IL, USA) was used for statistical analysis and GraphPad Prism 9.0 (GraphPad Software Inc., CA, USA) was used to produce the graphs.

**Figure 1:**
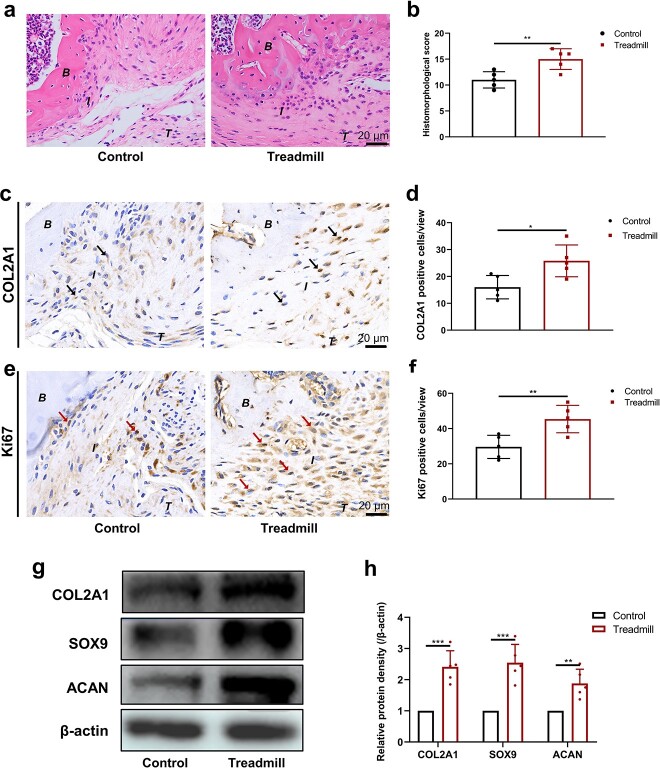
Treadmill training improves tendon–bone insertion healing and fibrochondrocyte proliferation. (**a**, **b**) Typical hematoxylin–eosin staining images and histomorphological scores in the control and treadmill groups. (**c**, **d**) Typical Col2a1 immunohistochemical image and the Col2a1-positive cell ratio in the control and treadmill groups. (**e**, **f**) Representative immunohistochemical staining patterns of Ki67 and the Ki67-positive cell ratio in the control and treadmill groups. (**g**, **h**) Col2a1, Sox9 and Acan protein expression levels were examined using western blotting after treadmill training. The results are presented as the mean ± SD, n = 5. Scale bar: 20 μm. ^*^p< 0.05, ^*^^*^p < 0.01, ^*^^*^^*^p < 0.001. *B* bone, *I* tendon–bone insertion, *T* tendon, *Col2a1* collagen, type II alpha 1, *Sox9* SRY-box transcription factor 9, *Acan* aggrecan

**Figure 2:**
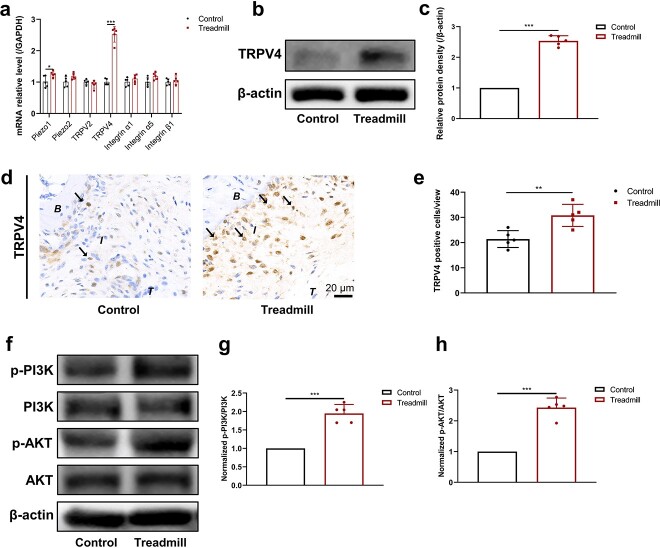
Treadmill training activates the TRPV4-PI3K/AKT signaling pathway during tendon–bone insertion healing. (**a**) mRNA expression levels of Piezo1, Piezo2, TRPV2, TRPV4, integrin α1, integrin α5 and integrin β1 in the control and treadmill groups. (**b**, **c**) Protein levels of TRPV4 in the control and treadmill groups were examined using western blotting. (**d**, **e**) Representative immunohistochemical staining images for TRPV4 and the proportion of TRPV4-positive cells in the control and treadmill groups. (**f–h**) Expression of the p-PI3K, PI3K, p-AKT and AKT proteins, as revealed by western blotting and normalized to the p-PI3K/PI3K and p-AKT/AKT ratios after treadmill training. The results are presented as the mean ± SD, n = 5. Scale bar: 20 μm. ^*^ *p*< 0.05, ^*^^*^p < 0.01, ^*^^*^^*^p < 0.001. *B* bone, *I* tendon–bone insertion, *T* tendon, *GAPDH* glyceraldehyde-3-phosphate dehydrogenase, *TRPV2* transient receptor potential cation channel subfamily V member 2, *TRPV4* transient receptor potential cation channel subfamily V member 4, *PI3K* phosphoinositide 3-kinase, *p-PI3K* phosphorylated PI3K, *AKT* protein kinase B, *p-AKT* phosphorylated AKT

## Results

### Treadmill training improves TBI healing and fibrochondrocyte proliferation

In order to investigate whether treadmill training affected TBI healing, hematoxylin–eosin staining was carried out to evaluate histological manifestations (Figure 1a, b). The results showed that the histomorphological score of TBI healing was significantly greater in the treadmill group than in the control group according to the histomorphological scoring system for TBI healing (supplementary Table S2), indicating a better histological performance of TBI in the treadmill training group. The immunohistochemical staining results showed that the percentage of Col2a1-positive cells was significantly greater in the treadmill group (Figure 1c, d). Immunohistochemical staining was used to observe the change in fibrochondrocyte proliferation and showed a greater proportion of Ki67-positive fibrochondrocytes after treadmill training (Figure 1e, f). The western blotting results showed that Col2a1, Sox9 and Acan protein expression was significantly greater after treadmill training (Figure 1g, h), suggesting that treadmill training could promote fibrochondrocyte proliferation during the TBI healing process.

**Figure 3:**
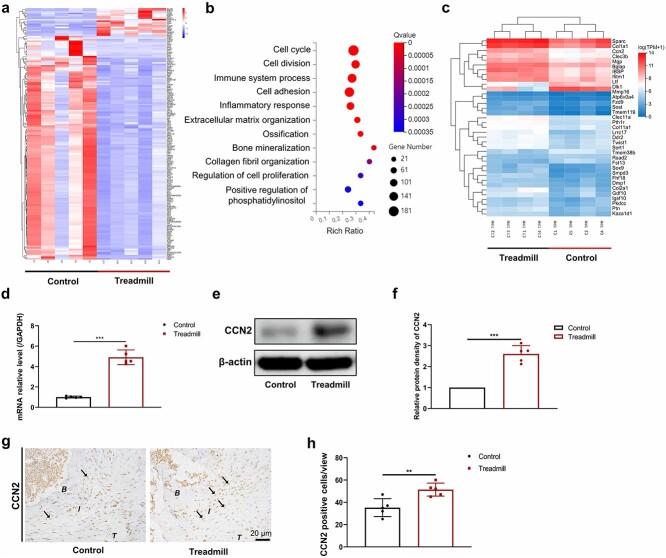
CCN2 may be a key molecule in the promotion of tendon–bone insertion healing by treadmill training. (**a–c)** A total of 2371 differentially expressed genes were selected by transcriptome sequencing. Gene ontology functional enrichment analysis was subsequently performed on the differentially expressed genes and the target genes were determined via a cluster analysis heatmap. (**d**) mRNA expression levels of CCN2 after treadmill training. (**e**, **f**) Protein levels of CCN2 were detected using western blotting after treadmill training. (**g**, **h**) Representative immunohistochemical staining images of CCN2 and the proportion of CCN2-positive cells after treadmill straining. Results are presented as the mean ± SD, n = 5. Scale bar:  20 μm. ^*^^*^p < 0.01, ^*^^*^^*^p < 0.001. *B* bone, *I* tendon–bone insertion, *T* tendon, *GAPDH* glyceraldehyde-3-phosphate dehydrogenase, *CCN2* cellular communication network factor 2

### Treadmill training activates the TRPV4-PI3K/AKT signaling pathway during TBI healing

Subsequently, we observed the effect of treadmill training on local cellular mechanoreceptor expression in the TBI, and the RT-PCR results showed that TRPV4 expression was significantly greater than that of other mechanoreceptors (Figure 2a). To verify the effect of treadmill training on TRPV4 expression, we performed western blotting and immunohistochemical staining, and the results showed that the TRPV4 protein expression level was significantly increased in the treadmill training group (Figure 2b, c). Immunohistochemical staining also showed that the percentage of TRPV4-positive cells was significantly increased after treadmill training (Figure 2d, e). In addition, we observed the effect of treadmill training on the downstream proliferation signaling pathway of TRPV4. The PI3K/AKT signaling pathway is an important regulator of cellular proliferation in fibrochondrocytes. The western blotting results showed that the protein expression of p-PI3K and p-AKT increased significantly after treadmill training, while there was no significant difference in the protein expression of PI3K and AKT (Figure 2f–h). The above results suggest that treadmill training may regulate fibrochondrocyte proliferation by promoting TRPV4 expression and activating the PI3K/AKT signaling pathway.

**Figure 4:**
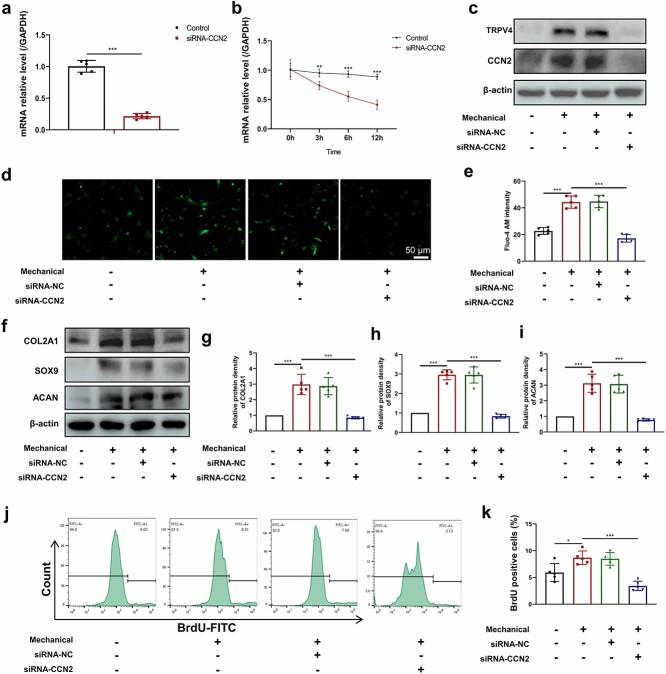
Downregulation of CCN2 inhibits TRPV4-induced Ca^2+^ activation and proliferation of fibrochondrocytes *in vitro*. (**a–c**) Validation of the efficacy of siRNA-CCN2. (a) siRNA-CCN2 significantly reduced CCN2 mRNA expression levels. (b) siRNA-CCN2 accelerated TRPV4 mRNA degradation after adding actinomycin D for 3 6 and 12 h. (c) Western blot analysis showed that siRNA-CCN2 significantly downregulated TRPV4 protein expression. (**d**, **e)** Fluorescence image and intensity of Fluo4-AM of fibrochondrocytes treated with mechanical stimulation and siRNA-CCN2. (**f–i**) Effect of mechanical stimulation and siRNA-CCN2 on the protein expression of Col2a1, Sox9 and Acan in fibrochondrocytes. (**j**, **k**) Fibrochondrocytes were treated with mechanical stimulation and siRNA-CCN2, and flow cytometry was used to determinate the BrdU-positive fibrochondrocytes. Results are presented as the mean ± SD, n = 5. Scale bar: 50 μm. ^*^p < 0.05, ^*^^*^p < 0.01, ^*^^*^^*^p < 0.001. *GAPDH g*lyceraldehyde-3-phosphate dehydrogenase, *CCN2* cellular communication network factor 2, *TRPV4* transient receptor potential cation channel subfamily V member 4, *NC* negative control, *Col2a1* collagen type II alpha 1, *Sox9* SRY-box transcription factor 9, *Acan* aggrecan, *BrdU* bromodeoxyuridine

### C‌CN2 may be a key molecule in the promotion of TBI healing by treadmill training

To elucidate the specific mechanism by which treadmill training promotes TBI healing and fibrochondrocyte proliferation, we sequenced the transcriptomes of the TBI tissues in the treadmill and control groups; a quality control map of the transcriptome sequencing data is shown in supplementaryFigure S2 (see online supplementary material). A total of 2731 differentially expressed genes were observed in the runner training group compared to the control group, of which 1186 were upregulated including TRPV4 (Figure S5) and 1545 were downregulate (Figure 3a, Figure S2). We performed gene ontology functional enrichment analysis on these 2731 differentially expressed genes and found that the cell cycle and other pathways were significantly enriched; interestingly, we found that ossification, a pathway closely related to fibrocartilage layer formation, was also significantly enriched (Figure 3b). We further analyzed the enriched pathways related to ossification and a heatmap of the cluster analysis results was generated to show the differential expression of the genes enriched in this pathway. By analyzing the expression of these genes and combining the results with those in the literature, we found that CCN2 may play an important role (Figure 3c). Next, RT-PCR (Figure 3d), western blotting (Figure 3e, f) and immunohistochemical staining (Figure 3g, h) were used to confirm that the treadmill training group had significantly greater CCN2 expression.

**Figure 5:**
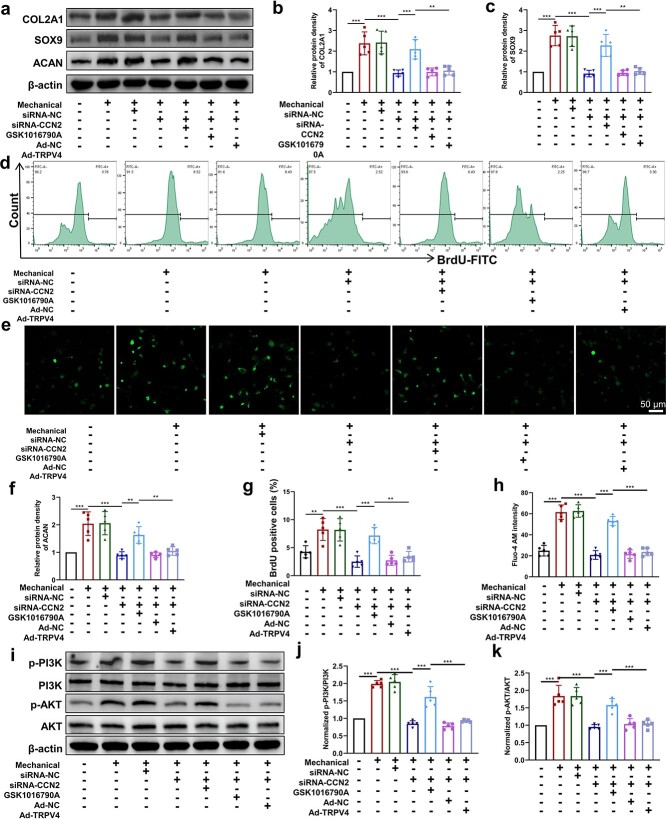
CCN2 promotes fibrochondrocyte proliferation by maintaining TRPV4 activity and the PI3K/AKT signaling pathway *in vitro*. (**a–d**) Protein levels of Col2a1, Sox9 and Acan in fibrochondrocytes treated with mechanical stimulation, siRNA-CCN2, 10 nM GSK1016790A or Ad-TRPV4 were analyzed using western blotting. (**e**, **f**) Flow cytometry assay was performed to measure the proliferative capacities of fibrochondrocytes. Fibrochondrocytes were subjected to mechanical stimulation, siRNA-CCN2, 10 nM GSK1016790A or Ad-TRPV4. (**g**, **h**) Fibrochondrocytes were subjected to mechanical stimulation, siRNA-CCN2, 10 nM GSK1016790A or Ad-TRPV4, and changes in intracellular calcium levels were measured using confocal microscopy. (**i–k**) Fibrochondrocytes were subjected to mechanical stimulation, siRNA-CCN2, 10 nM GSK1016790A or Ad-TRPV4, and the protein expression levels of p-PI3K, PI3K, p-AKT and AKT were examined by western blot analysis. The results are presented as the mean ± SD, n = 5. Scale bar: 50 μm. ^*^^*^p < 0.01, ^*^^*^^*^p < 0.001. *Col2a1* collagen type II alpha 1, *Sox9* SRY-Box transcription factor 9, *Acan* aggrecan, *NC* negative control, *CCN2* cellular communication network factor 2, *TRPV4* transient receptor potential cation channel subfamily V member 4, *BrdU* bromodeoxyuridine, *PI3K* phosphoinositide 3-kinase, *p-PI3K* phosphorylated PI3K, *AKT* protein kinase B, *p-AKT* phosphorylated AKT

**Figure 6:**
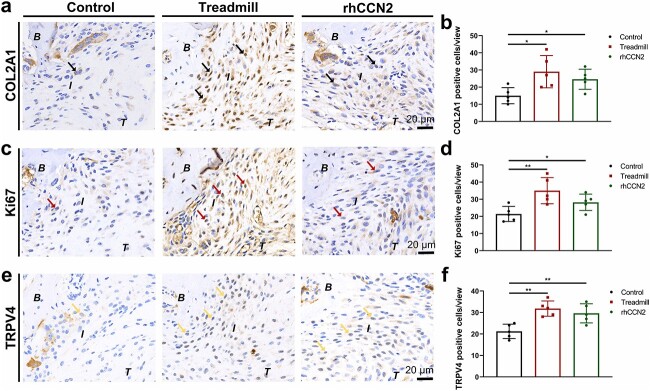
rhCCN2 promotes fibrochondrocyte TRPV4 expression and proliferation during TBI healing. (**a**, **b**) Representative images of Col2a1 immunohistochemical staining and the proportion of Col2a1-positive cells after treadmill training and rhCCN2 treatment. (**c**, **d**) Ki67 immunohistochemical staining was performed to observe proliferation and the proportion of Ki67-positive fibrochondrocytes. (**e**, **f**) Typical immunohistochemical staining of TRPV4 and the proportion of TRPV4-positive cells. Arrows in a: Col2a1-positive cells; Arrows in c: Ki67-positive cells; Arrows in e: TRPV4-positive cells. The results are presented as the mean ± SD, n = 5. Scale bar:  20 μm. ^*^p < 0.05, ^*^^*^p < 0.01. *B b*one, *I* tendon–bone insertion, *T* tendon, *Col2a1* collagen type II alpha 1, *TRPV4* transient receptor potential cation channel subfamily V member 4, *rhCCN2* recombinant human CCN2

### Downregulation of CCN2 inhibits TRPV4 activation and the proliferation of fibrochondrocytes *in vitro*

To investigate how CCN2 and TRPV4 interact, we used primary fibrochondrocytes *in vitro*. First, our RT-PCR results showed that fibrochondrocyte CCN2 mRNA expression was significantly reduced after siRNA-CCN2 treatment (Figure 4a). TRPV4 mRNA stability was observed after the addition of actinomycin D (10 mg/ml), and the results showed that 3, 6 and 12 h after treatment, siRNA-CCN2-treated TRPV4 mRNA levels were significantly lower than those in the control group (Figure 4b). Then, we evaluated the effect of siRNA-CCN2 on TRPV4 protein expression and function in fibrochondrocytes. Western blot analysis and calcium fluorescence imaging revealed that mechanical stimulation promoted TRPV4 protein expression and calcium influx; however, TRPV4 protein expression was significantly reduced (Figure 4c) and the Fluo-4 AM fluorescence intensity was diminished (Figure 4d, e) after siRNA-CCN2 treatment. To verify the effects of siRNA-CCN2 on fibrochondrocyte gene expression, proliferation and apoptosis under mechanically stimulated conditions, we first used western blotting to show that siRNA-CCN2 weakened the promotion of Col2a1, Sox9 and Acan expression by mechanical stimulation (Figure 4f–i). Our flow cytometry results showed that the percentage of BrdU-positive fibrochondrocytes was significantly increased after mechanical stimulation, while siRNA-CCN2 treatment reversed this effect (Figure 4j, k), and no significant difference in the fibrochondrocyte apoptosis rate was observed after mechanical stimulation or siRNA-CCN2 treatment (supplementary Figure S3a, b, see online supplementary material). The above results suggest that siRNA-CCN2 can impair the ability of mechanical stimulation to promote fibrochondrocyte proliferation and the protein expression and function of TRPV4 *in vitro*.

**Figure 7:**
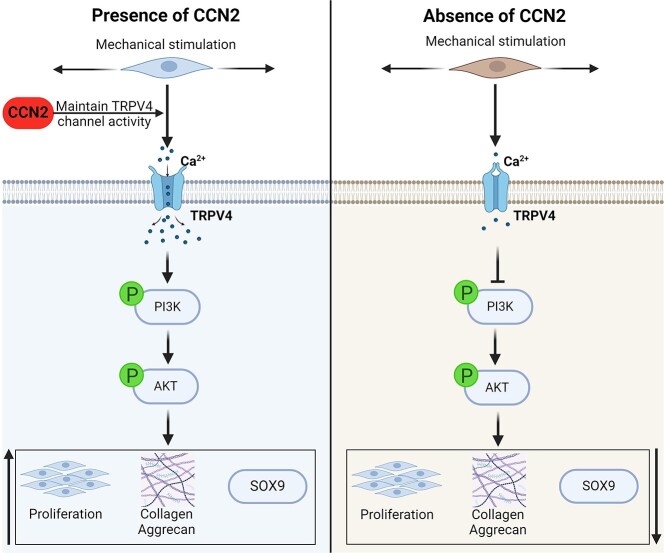
Molecular mechanisms by which CCN2 regulates the activity of TRPV4 channels under mechanical stimulation in fibrochondrocytes. Mechanical stimulation promotes TRPV4 protein expression and channel activation in fibrochondrocytes, and the presence of CCN2 can maintain the TRPV4 channel activation state and promote fibrochondrocyte proliferation through the downstream PI3K/AKT signaling pathway. *CCN2* cellular communication network factor 2, *TRPV4* transient receptor potential cation channel subfamily V member 4, *PI3K* phosphoinositide 3-kinase, *AKT* protein kinase B, *Sox9* SRY-box transcription factor 9

### C‌CN2 promotes fibrochondrocyte proliferation by maintaining TRPV4 activity and the PI3K/AKT signaling pathway *in vitro*

To determine whether CCN2 affects the fibrochondrocyte proliferative capacity via TRPV4, we treated fibrochondrocytes with the TRPV4 agonist GSK1016790A and the TRPV4 overexpression adenovirus (Ad-TRPV4) (Figure S1). First, western blot analysis revealed that adding GSK1016790A partially reversed the inhibitory effect of siRNA-CCN2 on fibrochondrocyte Col2a1, Sox9 and Acan protein expression, while Ad-TRPV4 did not significantly reverse this effect (Figure 5a–c and f). The flow cytometry results also showed that adding GSK1016790A partially restored the proliferation-promoting effect of mechanical stimulation on fibrochondrocytes, but this effect was not observed after Ad-TRPV4 treatment (Figure 5d and g). In addition, we performed confocal microscopy by adding the fluorescent calcium indicator Fluo-4-AM, and the fluorescence intensity analysis showed that the intracellular calcium-ion fluorescence intensity increased significantly after the addition of GSK1016790A to the fibrochondrocytes, while the fluorescence intensity was not significantly enhanced after Ad-TRPV4 treatment (Figure 5e and h). Finally, we observed changes in the PI3K/AKT signaling pathway downstream of TRPV4. Western blot results showed that p-PI3K and p-AKT protein expression was significantly increased after GSK1016790A treatment, while Ad-TRPV4 did not reverse the inhibitory effect produced by siRNA-CCN2, and the PI3K and AKT proteins were maintained at the same baseline (Figure 5i–k). Treatment of chondrocytes with 50 μmol of the PI3K pathway inhibitor LY294002 (MedChemExpress, USA) prior to mechanical stimulation and GSK1016790A treatment significantly inhibited the protein expression of Col2a1, Acan and Sox9 (supplementary Figure S4, see online supplementary material). The above results confirmed that CCN2 could enhance fibrochondrocyte proliferation by activating TRPV4 channels and promoting Ca^2+^ influx and PI3K/AKT signaling pathway activation under mechanical stimulation.

### rhCCN2 promotes fibrochondrocyte TRPV4 expression and proliferation during TBI healing

CCN2 acts as a mechanosensory regulator of fibrochondrocytes and we exogenously added the rhCCN2 protein during TBI healing. Immunohistochemical analysis revealed that the percentage of Col2a1-positive fibrochondrocytes treated with rhCCN2 protein was greater than that in the control group (Figure 6a, b), and the percentage of Ki67-positive fibrochondrocytes was significantly greater (Figure 6c, d). Additionally, the percentage of TRPV4-positive cells was significantly greater than that in the control group (Figure 6e, f), which could partially mimic the effect of treadmill training. Based on these results, we speculate that CCN2 may promote TBI healing and fibrochondrocyte proliferation by affecting TRPV4 expression.

## Discussion

The TBI is a complex and specialized area located at the interface between tendons and bones. Its main function is force transmission. Injury at the TBI is common and the recurrence of injuries is high, which is the main factor leading to a permanent inability to continue physical activity [33, 34]. TBI healing is difficult, the mechanism is complex, and the healing process is driven by a variety of coordinated biological and mechanical factors, of which mechanical factors play an important role [1]. The impact of mechanical stimulation on TBI healing has been controversial, and the main controversy is that the ideal timing of postoperative mechanical stimulation has not been elucidated [35, 36].

At present, mainstream research has indicated that short-term braking and delayed moderate mechanical stimulation after surgeryinjury can promote TBI healing, while either premature or excessive mechanical stimulation is not conducive to TBI healing [37, 38]. Lu *et al*. showed that starting low-intensity pulsed ultrasound treatment on postoperative day 7 was more conducive to recovery of the TBI microstructure, histology and mechanical strength than treatment started immediately postoperatively and on postoperative day 14 [39]. In this study, we selected treadmill training as a postoperative mechanical stimulation protocol and found that starting treadmill training at postoperative day 7 could lead to a higher histomorphological score and greater TBI healing quality. In our recent study, the fibrocartilage complex was confirmed to be the key anatomic and functional structure of the TBI, while the maintenance of chondrocyte homeostasis and proliferative activity is essential for healing. Therefore, we further observed cartilage marker expression by western blotting, which revealed significantly increased expression of Col2a1, Sox9 and Acan in the treadmill training group, while immunohistochemical staining revealed increased expression of Col2a1-positive and Ki67-positive fibrochondrocytes at the interface of the TBI. We used immunohistochemistry to clarify that treadmill training promoted the repair of the fibrocartilage complex by promoting the proliferation of fibrochondrocytes, but the underlying mechanism remains unclear.

Mechanoreceptors are effector proteins that chondrocytes use to recognize and respond to mechanical stimulation, transforming extracellular mechanical stimulation signals into intracellular biochemical signals and regulating chondrocyte growth, differentiation and proliferation [40]. To clarify how mechanical stimulation from treadmill training affects the proliferation of fibrochondrocytes, we used PCR to examine the mRNA expression of common mechanoreceptor genes on fibrochondrocytes. The results showed that TRPV4 of the TRP family was most significantly elevated after treadmill training. According to previous reports, TRPV4 is closely associated with the development of chondrocytes. Willard *et al*. indicated that TRPV4 servesserved both as a marker and a regulator of induced pluripotent stem cell chondrogenesis [16]. Experiments by Muramatsu *et al*. reported that activating TRPV4 increased Sox9 mRNA and protein expression levels, indicating that it could regulate the Sox9 pathway, which is involved in chondrogenesis [15]. O’Conor *et al*. demonstrated that TRPV4 activation was a key mechanical signaling mechanism for regulating extracellular matrix biosynthesis in articular chondrocytes [17]. Through western blotting and immunohistochemical staining, we found that the TRPV4 protein was significantly increased after treadmill training, suggesting that TRPV4 may act as a transducer for treadmill training to promote fibrochondrocyte proliferation during TBI healing. We further examined the PI3K-AKT pathway, which was closely associated with chondrocyte proliferation [41], by performing western blotting at the protein level, and found that its phosphorylation levels were significantly elevated after treadmill training. Combined with the elevated TRPV4 expression, these results suggest that TRPV4 mediates fibrochondrocyte proliferation induced by treadmill training and is associated with the PI3K-AKT pathway.

Previous studies have shown that chondrocytes can feel mechanical stimulation through two mechanisms: mechanical conduction in direct response to the mechanical stimulation of extracellular matrix deformation and the release of cytokines combined with targeted receptors to assist in mechanical conduction [42, 43]. Therefore, to further investigate how the mechanical stimulation caused by treadmill training activates TRPV4, we performed transcriptome sequencing of tissue samples from mice with TBI injury repair. Gene ontology analysis revealed significant enrichment of ossification and the cell cycle, which is consistent with the chondrocyte proliferation phenotype found by histology. In addition, we also found significant enrichment in ossification and bone mineralization, which are closely related to cartilage development. Our experiments also confirmed that CCN2 expression was elevated in the repaired tissue of the TBI treadmill training group at the histological level. The literature has reported that CCN2 is an important component of the extracellular matrix, which has a multifunctional role in cellular proliferation, differentiation, extracellular matrix synthesis and tissue regeneration. More importantly, a review by Nishida and Kubota showed that CCN2 was similar to chondrocyte biomarkers that sense appropriate mechanical stimulation while simultaneously acting as mechanosensing regulators of chondrocyte differentiation [23]. Nishida *et al*. found that CCN2 was able to regulate chondrocyte integrin α5 expression and extracellular matrix production and was able to regulate the signaling pathway mediated by integrin α5 [24]. Gao and Brigstock reported that the integrin α5β1 was a direct binding receptor for CCN2 that participates in regulation of the migration, proliferation and adhesion of pancreatic stellate cells [44]. In addition, in the integrin family expressed in chondrocytes, CCN2 can also act as a ligand, binding to the integrins α6β1 and αvβ5 to regulate the cellular effects generated by mechanical stimulation [45, 46]. Another study showed that low-intensity pulsed ultrasound can promote CCN2 and cartilage marker expression by activating the influx of TRPV4 channel Ca^2+^ and activating the mitogen-activated protein kinase (MAPK) signaling pathway; simultaneously, CCN2 can also stabilize TRPV4 mRNA expression [24]. Thus, we speculate that by acting as a regulator of chondrocyte mechanosensing, CCN2 similarly regulates TRPV4 mechanosensory transduction in chondrocytes, but there is no evidence that CCN2 can regulate TRPV4 in mechanical stimulation conduction during TBI healing.

To clarify how CCN2 regulates TRPV4 perception of mechanical stimulation and thus fibrochondrocyte proliferation, we conducted *in vitro* experiments using primary fibrochondrocytes from TBI mice. We first administered siRNA-CCN2 on the basis of the mechanical stimulation of primary fibrochondrocytes and found that fibrochondrocyte TRPV4 protein expression was significantly decreased, and along with decreased calcium fluorescence intensity, it decreased cartilage marker expression and decreased proliferative capacity. This result suggested that siRNA-CCN2 could inhibit TRPV4 protein expression and channel activation and the proliferation capacity of fibrochondrocytes. On this basis, to determine how CCN2 affected TRPV4 in regulating fibrochondrocyte proliferation, we administered an agonist of TRPV4 (GSK1016790A) and Ad-TRPV4 [47]. The results indicated that GSK1016790A treatment partially restored the impact of mechanical stimulation on fibrochondrocyte proliferation as well as the calcium fluorescence intensity. However, Ad-TRPV4 treatment significantly increased the protein level of TRPV4 but did not have a similar effect to GSK1016790A. These results indicate that CCN2 promotes fibrochondrocyte proliferation by affecting TRPV4 channel activation. Therefore, given the reported interaction of the CCN2 protein with TRPV4 mRNA [48], whether and how this association regulates alternative splicing in addition to the stability of TRPV4 mRNA [49, 50], and subsequently permits the synthesis of effective/active TRPV4, should be further investigated. We also confirmed the involvement of the PI3K/AKT signaling pathway (Figure 7). Finally, to verify that the regulatory effect of CCN2 was also present *in vivo*, we found that local injection of rhCCN2 during the repair of the TBI in mice significantly promoted the proliferation of fibrochondrocytes and thus promoted TBI healing.

This study has the following limitations. First, although we confirmed that CCN2 can affect TRPV4 channel activity, we did not confirm how CCN2 affects TRPV4 activation or whether it can function as a novel ligand for TRPV4. Second, we only validated the effect of CCN2 on TRPV4 *in vitro*, while the *in vivo* evidence was relatively insufficient. Nevertheless, we demonstrated for the first time that CCN2 can participate in maintaining TRPV4 activity and enrich the mechanism by which fibrochondrocytes are receptive to mechanical stimulation.

## Conclusions

In summary, our study revealed that mechanical stimulation promotes Ca^2+^ influx through TRPV4 channels and activates the PI3K/AKT signaling pathway, accompanied by increased expression of the CCN2 protein, which may be a key regulatory protein in maintaining TRPV4 activation, thereby promoting fibrochondrocyte proliferation and TBI healing. Our findings have some guiding implications for new clinical physical therapy programs and drug development in the future.

## Abbreviations

Acan: Aggrecan; Ad-TRPV4: Adenovirus TRPV4; CCN2: Cellular communication network factor 2; Col2a1: Collagen type II alpha 1; NC: Negative control; p-AKT: Phosphorylated protein kinase B; p-PI3K: Phosphorylated phosphoinositide 3-kinase; rhCCN2: Recombinant human CCN2; RT-PCR: Real-time polymerase chain reaction; Sox9: SRY-box transcription factor 9; TBI: Tendon–bone insertion; TRPV4: Transient receptor potential ion-channel subfamily V member 4.

## Funding

This work was supported by the National Natural Science Foundation of China (NSFC, Nos. 82130071 and 82072516).

## Authors’ contributions

Conceptualization, Xuting Bian, Jindong Tan, Kanglai Tang and Lin Guo; Methodology, Yunjiao Wang and Xuting Bian; Software, Rui Wang and Shibo Xu; Validation, Lin Ma; Formal Analysis, Xiao Liu and Mei zhou; Investigation, Hong Tang and Gang He; Resources, Kanglai Tang and Lin Guo; Data Curation, Xiao Liu; Writing—Original Draft Preparation, Xuting Bian, Jindong Tan and Xiao Liu; WritingReview & Editing, Kanglai Tang and Lin Guo; Visualization, Yunjiao Wang; Supervision, Lin Ma and Gang He; Project Administration, Xuting Bian and Jindong Tan; Funding Acquisition, Kanglai Tang and Lin Guo.

## Data availability

The original results presented in the study are included in the article/supplementary material; further inquiries can be directed to the corresponding authors.

## Ethics approval and consent to participate

The animal study was reviewed and approved by the Animal Ethics Committee of the Army Medical University (AMUWEC20210782).

## Conflict of interest

None declared.

## Supplementary Material

Supplementary_material_5_7_tkae028
