# Supplementary material for: Mechanical stimulation promotes fibrochondrocyte proliferation by activating the TRPV4 signaling pathway during tendon–bone insertion healing: CCN2 plays an important regulatory role
Source: Burns Trauma. 2024 Oct 19;12:tkae028. doi: 10.1093/burnst/tkae028 (PMC11491146; doi:10.1093/burnst/tkae028)
Supplement: Supplementary_material_5_7_tkae028 [file supplementary_material_5_7_tkae028.doc]

**Mechanical stimulation promotes fibrochondrocyte proliferation via activating TRPV4 signaling pathway during tendon-bone insertion healing: CCN2 plays an important regulatory role**

Xuting Bian^1^, Xiao Liu^1^, Mei Zhou^1^, Hong Tang^1^, Rui Wang^2^, Lin Ma^1^, Gang He^1^, Shibo Xu^1^, Jindong Tan^1^, Kanglai Tang^1^* and Lin Guo^1^*

**Corresponding authors**

Kanglai Tang

State Key Laboratory of Trauma, Burn and Combined Injury, Department of Orthopaedics/Sports Medicine Center, Southwest Hospital, Army Medical University, Gaotanyan Street 30, Shapingba District, Chongqing 400038, China

Lin Guo

State Key Laboratory of Trauma, Burn and Combined Injury, Department of Orthopaedics/Sports Medicine Center, Southwest Hospital, Army Medical University, Gaotanyan Street 30, Shapingba District, Chongqing 400038, China

Email: guolin6212@163.com

**This additional file includes:**

Table S1 Primers and sequences used in this study

Table S2 Histomorphological scoring system for tendon-bone insertion healing

Table S3 Details of materials

Table S4 Genes list of Fig 3A

Fig. S1 Validation of TRPV4 overexpression with recombinant adenovirus transfection

Fig. S2 Quality control map of transcriptome sequencing

Fig. S3 siRNA-CCN2 has no significant effect on fibrochondrocytes apoptosis

Fig. S4 LY294002 inhibits the facilitative effect of mechanical stimulation on chondrocyte marker protein expression

Fig. S5 The statistical data illustrating the expression changes of TRPV4 from the transcriptome sequencing

| **Supplementary Table S1 Primers and sequences used in this study** | | |  |
| --- | --- | --- | --- |
| **Primer** | Forward Primer (5’-3’) | Reverse Primer (5’-3’) | |
| Mouse | | | |
| GAPDH | AGGTCGGTGTGAACGGATTTG | TGTAGACCATGTAGTTGAGGTCA | |
| CCN2 | GGGCCTCTTCTGCGATTTC | ATCCAGGCAAGTGCATTGGTA | |
| COL2A1 | GGGAATGTCCTCTGCGATGAC | GAAGGGGATCTCGGGGTTG | |
| SOX9 | GAGCCGGATCTGAAGAGGGA | GCTTGACGTGTGGCTTGTTC | |
| Aggrecan | CCTGCTACTTCATCGACCCC | AGATGCTGTTGACTCGAACCT | |
| TRPV2 | TGCTGAGGTGAACAAAGGAAAG | TCAAACCGATTTGGGTCCTGT | |
| TRPV4 | ATGGCAGATCCTGGTGATGG | GGAACTTCATACGCAGGTTTGG | |
| Piezo1 | TCATCATCCTTAACCACATGGTG | TGAAGACGATAGCTGTCATCCA | |
| Piezo2 | AGAGTCGGAAAAGAGATACCCTC | CCAGACGATACAGATGAGAAGGA | |
| Integrin α1 | CCTTCCCTCGGATGTGAGTCA | AAGTTCTCCCCGTATGGTAAGA | |
| Integrin α5 | CTTCTCCGTGGAGTTTTACCG | GCTGTCAAATTGAATGGTGGTG | |
| Integrin β1 | GAAGTGCCACCTCGTGTGAA | GGACCGTGGATTGCCAAAGT | |

|  | **Supplementary Table S2 Histomorphological scoring system for tendon-bone insertion healing**   |  | | --- | | | | | | |  |
| --- | --- | --- | --- | --- | --- | --- | --- |
| Items | | | Score criteria | | | | |
|  |  |  | 0 | 1 | 2 | 3 | |
| Tendon-to-bone interface | | Fibrocartilage cell number^a^ | 0%~25% | 25%~50% | 50%~75% | ≥75.0% | |
|  |  | Fibrocartilage cell alignment | None | Unorganized | Moderate alignment | Highly aligned | |
|  |  | Collagen fibre  continuity^a^ | 0%~25% | 25%~50% | 50%~75% | 75%~100% | |
|  |  | Collagen fibre  orientation^a^ | 0%~25% | 25%~50% | 50%~75% | 75%~100% | |
|  |  | Tidemark^a^ | 0%~25% | 25%~50% | 50%~75% | 75%~100% | |
| Cellularity^a^ | | | >400% | 300~400 | 200~300 | <200 | |
| Vascularity^b^ | | | >15 | 10~15 | 6~10 | ≤5 | |
| Inflammation | | | Abundant inflammatory cells | Moderate inflammatory cells | Minimal inflammatory cells | No inflammatory cells | |
| Total scores | | | 0~24 | | | | |

a:The percentage is the relative value compared with the uninjured tendon bone interface tissue.

b:Number of blood vessels per low power field (×10) from each section.

| | **Supplementary Table S3 Details of materials** | | --- | |
| --- | --- |

| Materials | Brand or manufacturer |
| --- | --- |
| C57 BL/6 mice | Byrness Weil biotech Ltd, Chongqing, China |
| rhCCN2 | Med Chem Express, USA |
| stretch chamber | NEPA GENE, Japan |
| CCN2 siRNA and negative control (NC) siRNA | Shanghai GenePharma Co., Ltd. |
| GP-transfect-Mate | Zeta Life, USA |
| GSK1016790A | Med Chem Express, USA |
| confocal microscope | Zeiss, Germany |
| PrimeScript™ RT Master Mix | Takara, Japan |
| SYBR Green RT-PCR kit | Takara, Japan |
| T-PER tissue protein extraction reagent | Thermo Fisher Scientific Inc., USA |
| BCA protein analysis kit | Beyotime, China |
| SDS-PAGE protein loading buffer (5X) | Beyotime, China |
| PVDF membranes | Merck Millipore Ltd., Germany |
| rabbit anti-CCN2 | ab6992, Abcam, WB:1:1000, IHC: 1:500 |
| rabbit anti-TRPV4 | PA5-77319, Invitrogen, WB:1:1000, IHC: 1:500 |
| rabbit anti-Ki67 | 28074-1-AP, Proteintech, 1:500 |
| rabbit anti-COL2A1 | ab34712, Abcam, 1:1000 |
| rabbit anti-SOX9 | ab185966, Abcam, 1:1000 |
| rabbit anti-Aggrecan | NB100-74350, Novus, 1:1000 |
| rabbit anti-PI3K | ab191606, abcam, 1:1000 |
| rabbit anti-p-PI3K(Y607) | ab182651, abcam, 1:1000 |
| rabbit anti-AKT | 10176-2-AP, Proteintech, 1:1000 |
| rabbit anti-p-AKT(Ser473) | 28731-1-AP, Proteintech, 1:1000 |
| rabbit anti-β-actin | 81115-1-RR, Proteintech, 1:5000 |
| goat anti-rabbit IgG (H&L)-HRP conjugate | SA00001-2, Proteintech, 1:2000 |
| Super ECL Western Blotting Detection Kit | Advansta, USA |
| Hematoxylin-eosin | Solarbio, China |
| BrdU cell proliferation assayKit kit | BD Biosciences, USA |
| Annexin V-FITC/PI double-stained apoptosis detection kit | BestBio, China |

| | **Supplementary Table S4 Genes list of Figure 3a** | | --- | |
| --- | --- |

| **Number** | **Gene** | **Number** | **Gene** | **Number** | **Gene** | **Number** | **Gene** | **Number** | **Gene** | **Number** | **Gene** | **Number** | **Gene** |
| --- | --- | --- | --- | --- | --- | --- | --- | --- | --- | --- | --- | --- | --- |
| **1** | **Mup22** | **26** | **Nos2** | **51** | **Nrg1** | **76** | **Rhov** | **101** | **Pglyrp1** | **126** | **Celsr3** | **151** | **S100a9** |
| **2** | **Mup10** | **27** | **Acod1** | **52** | **Mefv** | **77** | **Clnk** | **102** | **Trem3** | **127** | **Gca** | **152** | **Padi4** |
| **3** | **Cntn2** | **28** | **Il17f** | **53** | **Spatc1** | **78** | **Usp44** | **103** | **Plk1** | **128** | **Jph3** | **153** | **Lcn2** |
| **4** | **Crygs** | **29** | **Draxin** | **54** | **Ifitm1** | **79** | **Glt1d1** | **104** | **Serpinb1a** | **129** | **Mrgpra2a** | **154** | **Ngp** |
| **5** | **Ankrd1** | **30** | **Ermn** | **55** | **Fcgr4** | **80** | **Trim30b** | **105** | **Prom1** | **130** | **Ceacam10** | **155** | **Gm5294** |
| **6** | **Gldn** | **31** | **Osm** | **56** | **Tnfsf14** | **81** | **Lipg** | **106** | **Stfa1** | **131** | **Ifitm6** | **156** | **Ppp1r42** |
| **7** | **Myoz2** | **32** | **H1f3** | **57** | **Cd300lf** | **82** | **Tcp11x2** | **107** | **Stfa2** | **132** | **Fpr2** | **157** | **Stfa3** |
| **8** | **Krtap19-2** | **33** | **Shcbp1l** | **58** | **Li18rap** | **83** | **Slfn4** | **108** | **Treml2** | **133** | **Chil1** | **158** | **Il27** |
| **9** | **Fosb** | **34** | **H3c3** | **59** | **Nlrp12** | **84** | **Prok2** | **109** | **Slfn1** | **134** | **Gpt1** | **159** | **A530032D15Rik** |
| **10** | **Chga** | **35** | **H2ac13** | **60** | **Ppp1r3d** | **85** | **Olfm4** | **110** | **Ttc21a** | **135** | **FPr1** | **160** | **Mapk13** |
| **11** | **C7** | **36** | **Asb16** | **61** | **Gpr84** | **86** | **Cldn15** | **111** | **Ly75** | **136** | **Foxd4** | **161** | **Fgf23** |
| **12** | **Epha5** | **37** | **Gm27021** | **62** | **Adora3** | **87** | **Plac8** | **112** | **Slc2a3** | **137** | **Mrgpra2b** | **162** | **Pla2g10** |
| **13** | **Fgf5** | **38** | **Jchain** | **63** | **Gm36079** | **88** | **Crtac1** | **113** | **Amer2** | **138** | **Siglece** | **163** | **Itgad** |
| **14** | **Mptx2** | **39** | **Derl3** | **64** | **Clec5a** | **89** | **Oas3** | **114** | **Ccno** | **139** | **A530064D06Rik** |  |  |
| **15** | **Cdh6** | **40** | **Mzb1** | **65** | **Tarm1** | **90** | **Slc22a20** | **115** | **Ipcef1** | **140** | **Hck** |  |  |
| **16** | **Aadacl4** | **41** | **Bhlha15** | **66** | **Hdc** | **91** | **Cfap57** | **116** | **4930438A08Rik** | **141** | **Cxcr2** |  |  |
| **17** | **Ces4a** | **42** | **Sycp2** | **67** | **Chrnd** | **92** | **Ubash3a** | **117** | **Ankrd22** | **142** | **9830107B12Rik** |  |  |
| **18** | **Awat1** | **43** | **Trem1** | **68** | **Gpr15** | **93** | **Cst7** | **118** | **Pilrb2** | **143** | **Mmp8** |  |  |
| **19** | **Prl2c3** | **44** | **Arg2** | **69** | **Gm38525** | **94** | **Nkg7** | **119** | **Sell** | **144** | **Mcemp1** |  |  |
| **20** | **Krt6b** | **45** | **Cftr** | **70** | **Gm9733** | **95** | **Lrr1** | **120** | **Ifnlr1** | **145** | **Parvg** |  |  |
| **21** | **Cstdc5** | **46** | **Nlrp6** | **71** | **F10** | **96** | **Cebpe** | **121** | **Sorl1** | **146** | **Rac2** |  |  |
| **22** | **Hcar2** | **47** | **AA467197** | **72** | **Slpi** | **97** | **Cstdc4** | **122** | **B430306N03Rik** | **147** | **Ncf4** |  |  |
| **23** | **Il1b** | **48** | **Olr1** | **73** | **Fgr** | **98** | **Ltb4r1** | **123** | **Pilra** | **148** | **Pram1** |  |  |
| **24** | **Saa3** | **49** | **Tnf** | **74** | **Hrh2** | **99** | **Chil5** | **124** | **Atp8b4** | **149** | **Pygl** |  |  |
| **25** | **Ccl4** | **50** | **Clec4e** | **75** | **Il1f9** | **100** | **Mmp25** | **125** | **Abca13** | **150** | **Hk3** |  |  |

**Supplementary Figure 1 Validation of TRPV4 overexpression with recombinant adenovirus transfection**


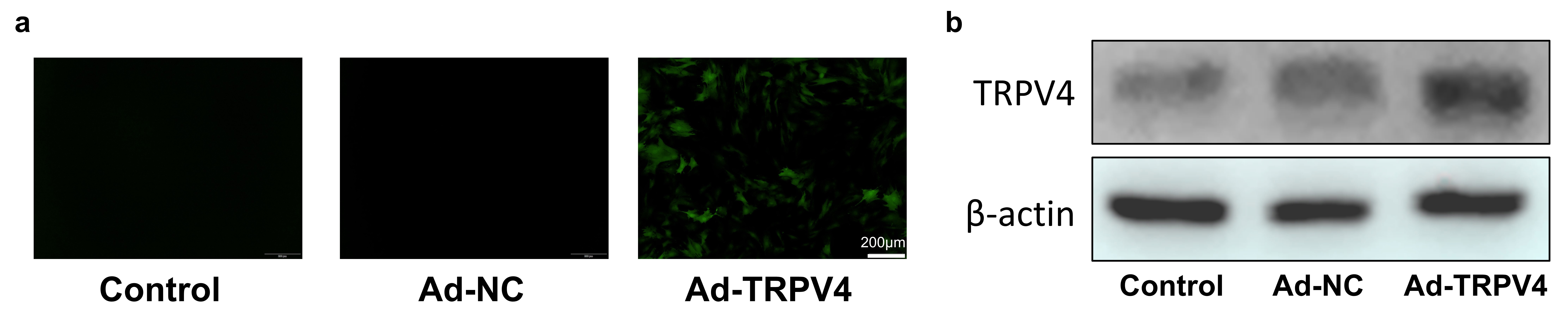


**Figure. S1 Validation of TRPV4 overexpression with recombinant adenovirus transfection. a** Fluorescent images of TRPV4 overexpression with recombinant adenovirus transfection. **b** Western Blot analysis showed that TRPV4 overexpression with recombinant adenovirus transfection significantly increases the protein expression level of TRPV4. Scale bar=200 μm. *NC* negative control, *TRPV4* transient receptor potential cation channel subfamily V member 4

**Supplementally Figure 2: Quality control map of transcriptome sequencing**


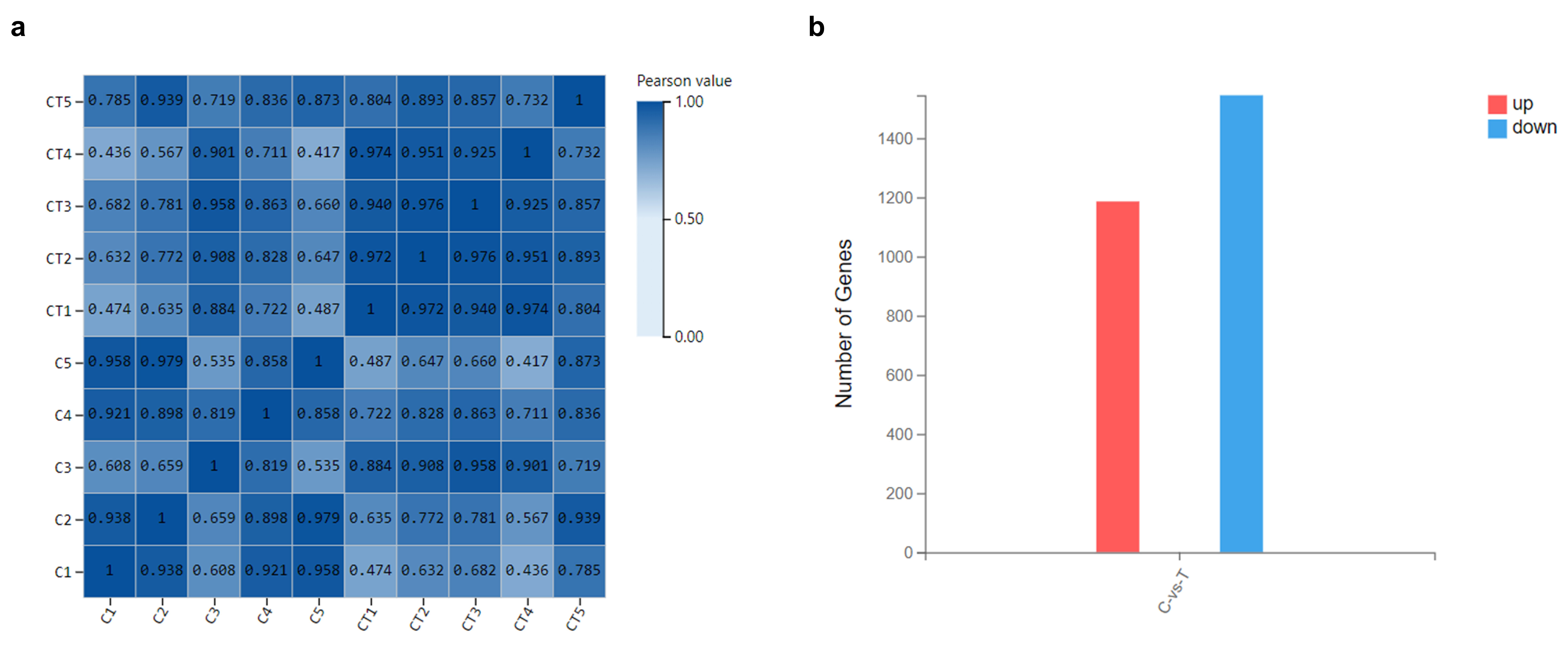


**Figure. S2 Quality control map of transcriptome sequencing. a** The correlation heatmap of transcriptome sequencing. **b** Differentially expressed gene count statistics for transcriptome sequencing. *C* control group, *CT* training group

**Supplementally Figure 3 siRNA-CCN2 has no significant effect on fibrochondrocytes apoptosis**


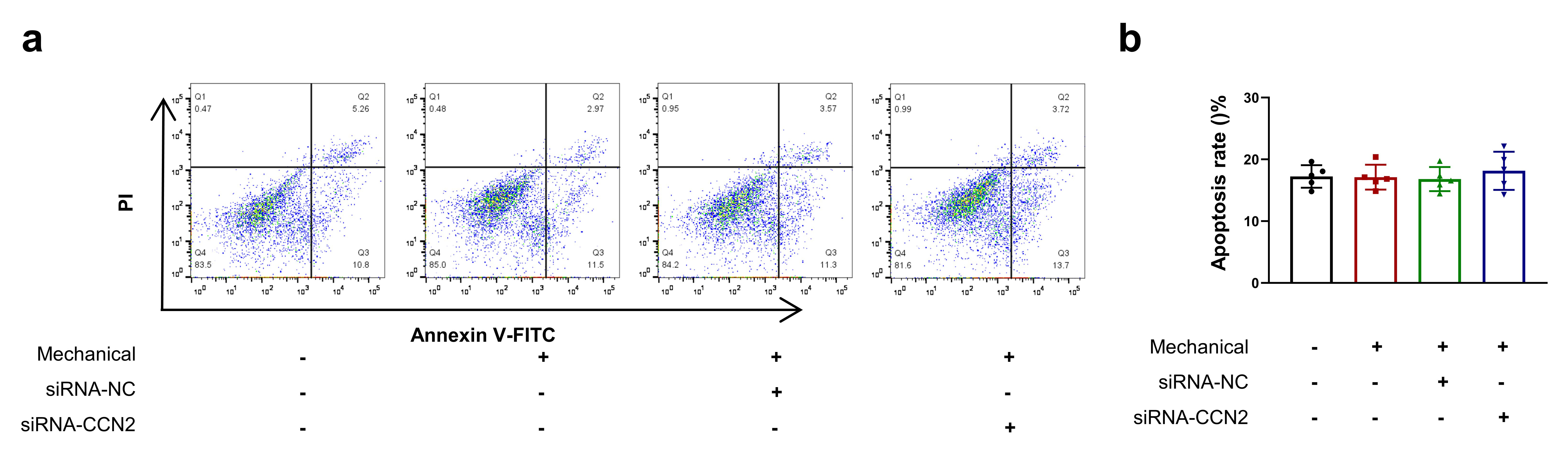


**Figure. S3 siRNA-CCN2 has no significant effect on fibrochondrocytes apoptosis. a** Typical flow cytometry image of Annexin V-FITC staining after mechanical stimulation and siRNA-CCN2 treatment. **b** Fibrochondrocytes apoptosis rate after mechanical stimulation and siRNA-CCN2 treatment. Results are presented as the mean ± SD, n=5. * *P*< 0.05, ** *P*< 0.01, *** *P*< 0.001. *NC* negative control, *CCN2* cellular communication network factor 2

**Supplementally Figure 4 LY294002 inhibits the facilitative effect of mechanical stimulation on chondrocyte marker protein expression**

Treatment of Chondrocyte with 50 μM PI3K pathway inhibitor LY294002 (Med Chem Express, USA) prior to stimulation with mechanical stimulation and GSK1016790A led to a significant inhibitory effects on protein expression levels of COL2A1, ACAN and SOX9.


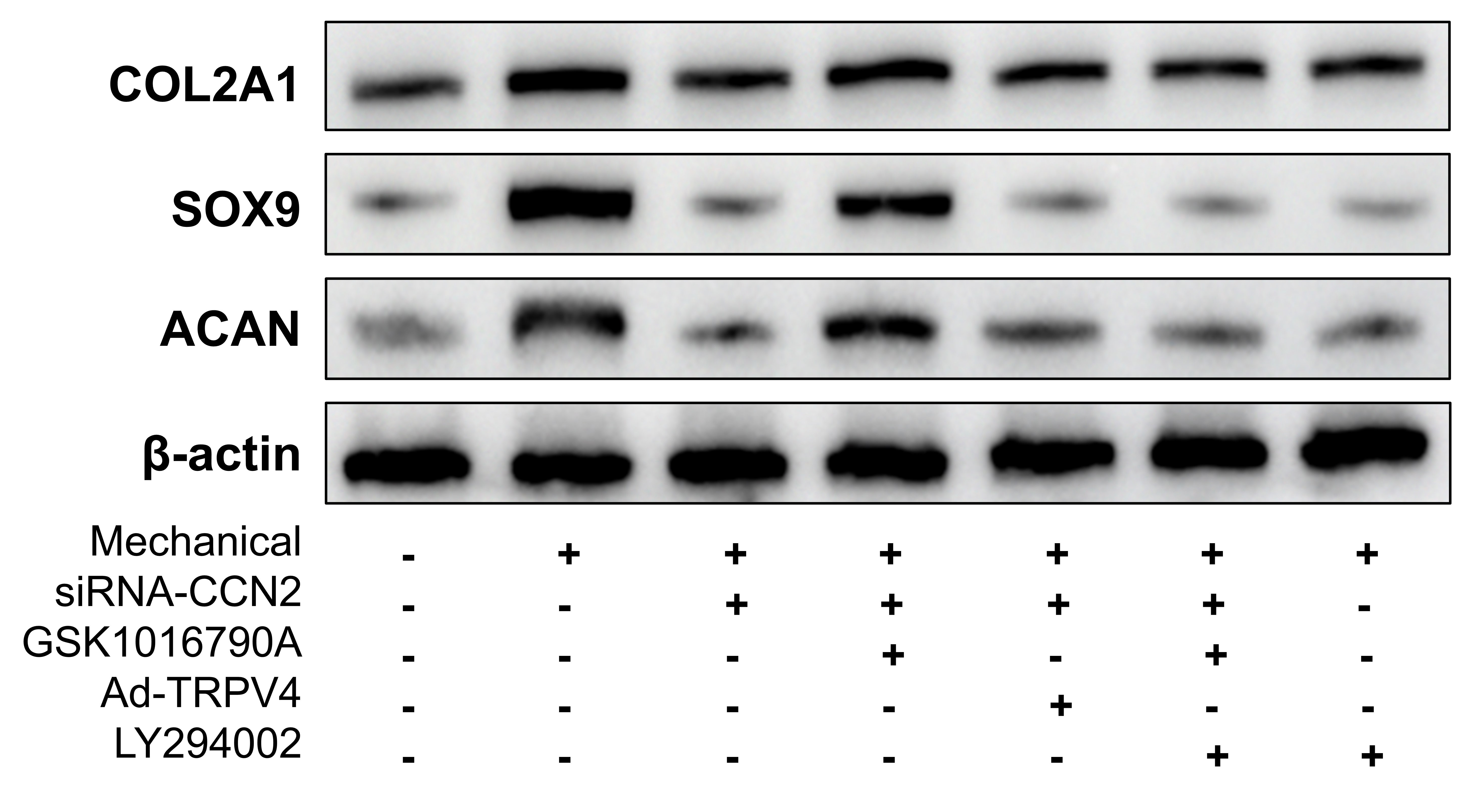


**Figure. S4 LY294002 inhibits the facilitative effect of mechanical stimulation on chondrocyte marker protein expression.** Representative Western Blot band images of COL2A1, ACAN and SOX9 in chondrocytes treated with mechanical stimulation, siRNA-CCN2, GSK1016790A, Ad-TRPV4 and LY294002. *COL2A1* collagen type II alpha 1, *SOX9* SRY-Box transcription factor 9, *ACAN* aggrecan, *CCN2* cellular communication network factor 2, *TRPV4* transient receptor potential cation channel subfamily V member 4

**Supplementally Figure 5 The statistical data illustrating the expression changes of TRPV4 from the transcriptome sequencing**


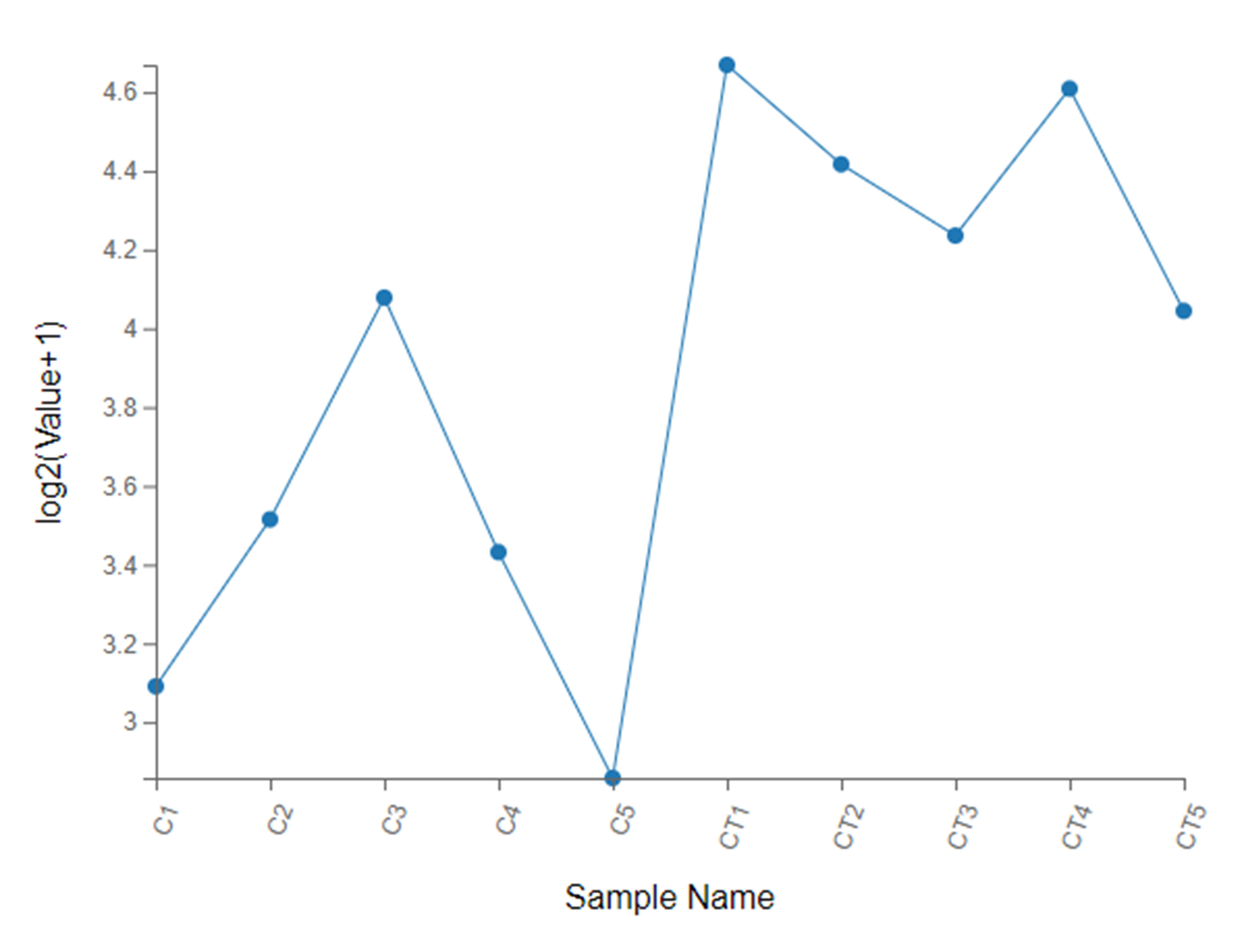


**Figure. S5 The statistical data illustrating the expression changes of TRPV4 from the transcriptome sequencing.** mRNA expression levels of TRPV4 of each group from the transcriptome sequencing. *C* control group, *CT* training group
